# Metabolic engineering of the 2-ketobutyrate biosynthetic pathway for 1-propanol production in *Saccharomyces cerevisiae*

**DOI:** 10.1186/s12934-018-0883-1

**Published:** 2018-03-09

**Authors:** Yuya Nishimura, Terumi Matsui, Jun Ishii, Akihiko Kondo

**Affiliations:** 1 Graduate School of Science, Technology and Innovation, Kobe University, 1-1 Rokkodai, Nada, Kobe, Japan; 2 Department of Chemical Science and Engineering, Graduate School of Engineering, Kobe University, 1-1 Rokkodai, Nada, Kobe, Japan

**Keywords:** 1-Propanol, Yeast, *S. cerevisiae*, Fermentation, 2-Ketobutyrate

## Abstract

**Background:**

To produce 1-propanol as a potential biofuel, metabolic engineering of microorganisms, such as *E. coli*, has been studied. However, 1-propanol production using metabolically engineered *Saccharomyces cerevisiae*, which has an amazing ability to produce ethanol and is thus alcohol-tolerant, has infrequently been reported. Therefore, in this study, we aimed to engineer *S. cerevisiae* strains capable of producing 1-propanol at high levels.

**Results:**

We found that the activity of endogenous 2-keto acid decarboxylase and alcohol/aldehyde dehydrogenase is sufficient to convert 2-ketobutyrate (2 KB) to 500 mg/L 1-propanol in yeast. Production of 1-propanol could be increased by: (i) the construction of an artificial 2 KB biosynthetic pathway from pyruvate via citramalate (*cimA*); (ii) overexpression of threonine dehydratase (*tdcB*); (iii) enhancement of threonine biosynthesis from aspartate (*thrA*, *thrB* and *thrC*); and (iv) deletion of the *GLY1* gene that regulates a competing pathway converting threonine to glycine. With high-density anaerobic fermentation of the engineered *S. cerevisiae* strain YG5C4231, we succeeded in producing 180 mg/L 1-propanol from glucose.

**Conclusion:**

These results indicate that the engineering of a citramalate-mediated pathway as a production method for 1-propanol in *S. cerevisiae* is effective. Although optimization of the carbon flux in *S. cerevisiae* is necessary to harness this pathway, it is a promising candidate for the large-scale production of 1-propanol.

**Electronic supplementary material:**

The online version of this article (10.1186/s12934-018-0883-1) contains supplementary material, which is available to authorized users.

## Background

As a means of mitigating environmental issues, such as global warming and the depletion of fossil fuels, biofuels and products from sustainable biomass resources have received significant attention in recent years. In particular, alcohols have been extensively studied, since they are already available as next-generation fuels and represent the building blocks of other chemicals. In this study, we focused on 1-propanol, which is generically used as a solvent and as a food additive, is found in paint and cosmetics, and is a chemical intermediate in the production of *n*-propylamine [1]. However, a natural microbial producer of 1-propanol has yet to be identified.

The budding yeast *Saccharomyces cerevisiae* is likely to be a good candidate for the production of 1-propanol. As *S. cerevisiae* has been used to produce ethanol, it is clear that the strain has tolerance to high concentrations of alcohols and other stresses during fermentation [2, 3], properties that should be useful for the industrial production of 1-propanol. Furthermore, *S. cerevisiae* strains that utilize not only glucose, but also xylose, as a sugar substrate have been developed to expand the range of applications of this biomass [4]. For these reasons, *S. cerevisiae* may be more suitable for the production of 1-propanol than other microbial hosts.

**Fig. 1:**
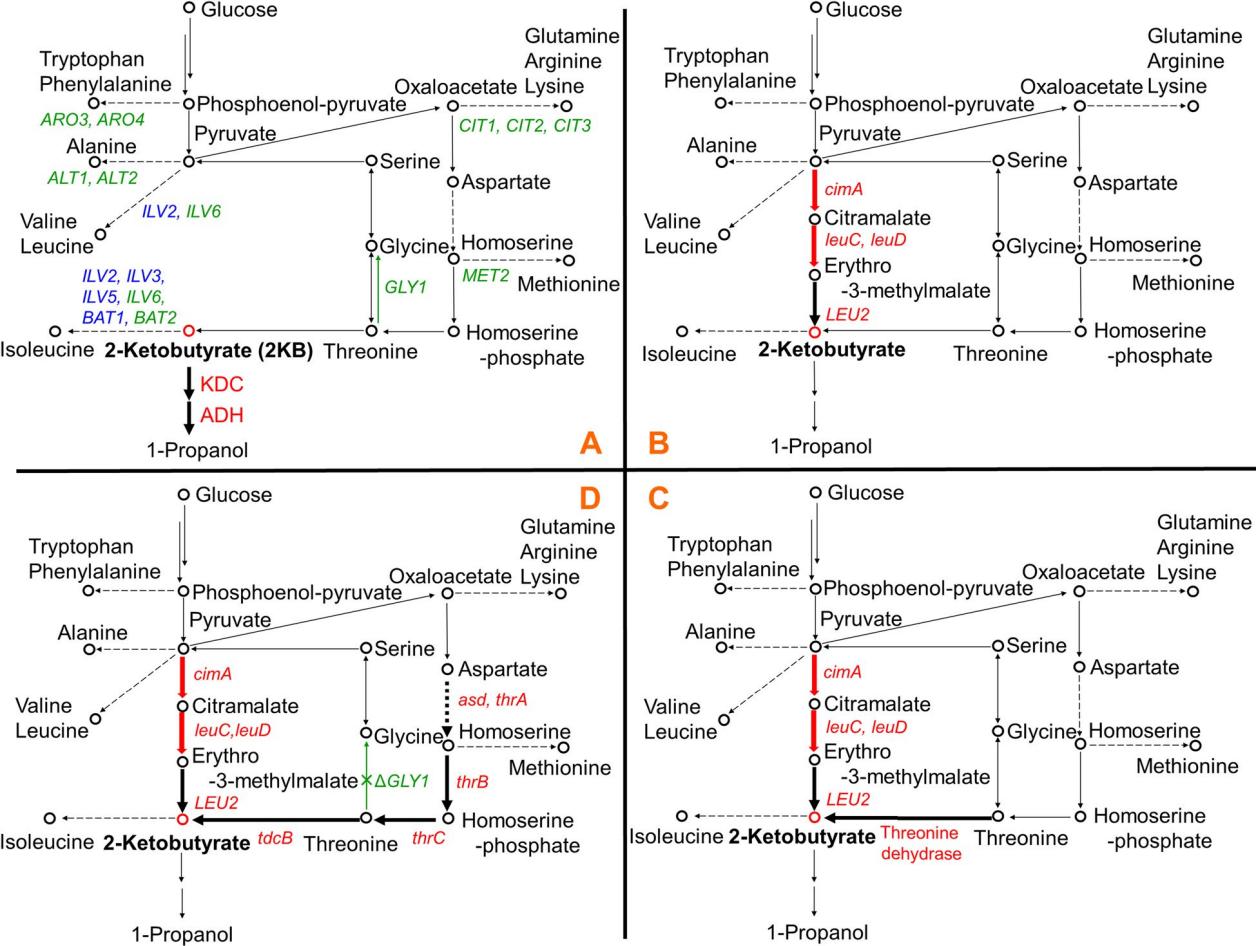
Pathways for 1-propanol production in *S. cerevisiae*. **a**–**d** show different theoretical methods to achieve production of this metabolite. Red letters indicate genes that are overexpressed. Blue and green letters indicate genes that are deleted. *ARO3* and *ARO4* encode 3-deoxy-d-arabino-heptulosonate-7-phosphate synthase. *ALT1* and *ALT2* encode alanine transaminase. *CIT1*, *CIT2* and *CIT3* encode citrate synthase. *MET2* encodes l-homoserine-*O*-acetyltransferase. *GLY1* encodes threonine aldolase. *ILV2 and ILV6* encode acetolactate synthase. *ILV3* encodes dihydroxyacid dehydratase. *ILV5* encodes acetohydroxyacid reductoisomerase. *BAT1* and *BAT2* encode branched-chain amino acid transaminase. *cimA* encodes citramalate synthase. *leuC* and *leuD* encode citramalate hydrolyase. *LEU2* encodes 3-isopropylmalate dehydrogenase. *asd* encodes aspartate-semialdehyde dehydrogenase. *thrA* encodes aspartokinase and homoserine dehydrogenase I. *thrB* encodes homoserine kinase. *thrC* encodes threonine synthase. *tdcB* encodes threonine dehydratase

Recently, it has been reported that the production of various alcohols from α-keto acids can be achieved using 2-keto acid decarboxylase (KDC) and alcohol/aldehyde dehydrogenase (ADH) in conjunction with metabolic engineering [5]. Using this method, the authors demonstrated the production of 2.1 mM of 1-propanol from 8 g/L (78.3 mM) of 2-ketobutyrate (2 KB) in *E. coli* [5]. It is also possible therefore, to convert 2 KB produced from threonine into 1-propanol by reaction with KDC and ADH in *S. cerevisiae* (Fig. 1a). *E. coli* primarily produce 2 KB from threonine; however, they can learn to produce 2 KB from pyruvate via citramalate once the citramalate synthase (*cimA*) gene is introduced. Using this approach, the production of up to 4.5 g/L of 1-propanol has been demonstrated in *E. coli* [6]. A similar strategy would be expected to produce 2 KB from pyruvate via citramalate in yeast, by introducing both citramalate synthase (*cimA*) and methylmalate dehydratase (*leuC* and *leuD*) and overexpressing isopropyl malate dehydrogenase (*LEU2*) (Fig. 1b).

Furthermore, others have reported that deleting the acetolactate synthase gene (*ILV2*), which directs a competing biochemical pathway, results in the production of 60 mg/L of 1-propanol in yeast [7]. Therefore, in the present study, we aimed to develop a metabolically engineered *S. cerevisiae* strain suitable for the production of 1-propanol utilizing a combination of these approaches, specifically the overexpression of genes for 1-propanol biosynthesis, together with deletion of the competing metabolic pathway.

## Methods

### Strains, plasmids, and primers

The yeast strains used in this study are listed in Table 1. *S. cerevisiae* YPH499 (MATa *ura3*-52 *lys2*-801 *ade2*-101 *trp1*-Δ63 *his3*-Δ200 *leu2*-Δ1, purchased from Stratagene, La Jolla, CA, USA) [8], BY4741 (MATa *his3*Δ1 *leu2*Δ0 *met15*Δ0 *ura3*Δ0) and the single gene deletion mutants (purchased from Thermo Scientific) [9] were used as yeast host strains. The plasmids and primers used in this study are summarized in Tables 2, 3 and Additional file 1, respectively. All plasmids were derived from the pGK and pATP vector series, in which gene expression is controlled either by the *PGK1* promoter, or the *ADH1*, *TDH1*, and *PGK1* promoters, respectively [10]. The *cimA*, *leuC* and *leuD* genes derived from *Methanocaldococcus jannaschii* were amplified from genomic DNA (NBRC No. 100440G, purchased from National Institute of Technology and Evaluation, Tokyo, Japan). The *leuC* and *leuD* genes derived from *Clostridium beijerinckii* were amplified from genomic DNA (NBRC No. 103909, purchased from National Institute of Technology and Evaluation, Tokyo, Japan). All other genes derived from *S. cerevisiae* and *E. coli* were amplified from YPH499 and BL21 (DE3) genomic DNA, respectively, using the primers shown in Table 3. The growth conditions, DNA techniques, and lithium-acetate method for transformations have been previously described [11, 12].

**Table 1:** Yeast strains constructed in this study

Strains	Genotypes
YPH499	MATa *URA3*-52 *LYS2*-801 *ADE2*-101 *TRP1*-Δ63 *HIS3*-Δ200 *LEU2*-Δ1
BY4741	MATa *HIS3*Δ1 *LEU2*Δ0 *MET15*Δ0 *URA3*Δ0
YA0K0	YPH499/pGK426/pGK423
YA0K1	YPH499/pGK426/pGK423-*kivd*
YA0K2	YPH499/pGK426/pGK423-*ARO10*
YA0K3	YPH499/pGK426/pGK423-*THI3*
YA1K0	YPH499/pGK426-*ADH1*/pGK423
YA1K1	YPH499/pGK426-*ADH1*/pGK423-*kivd*
YA1K2	YPH499/pGK426-*ADH1*/pGK423-*ARO10*
YA1K3	YPH499/pGK426-*ADH1*/pGK423-*THI3*
YA2K0	YPH499/pGK426-*ADH2*/pGK423
YA2K1	YPH499/pGK426-*ADH2*/pGK423-*kivd*
YA2K2	YPH499/pGK426-*ADH2*/pGK423-*ARO10*
YA2K3	YPH499/pGK426-*ADH2*/pGK423-*THI3*
YA3K0	YPH499/pGK426-*ADH5*/pGK423
YA3K1	YPH499/pGK426-*ADH5*/pGK423-*kivd*
YA3K2	YPH499/pGK426-*ADH5*/pGK423-*ARO10*
YA3K3	YPH499/pGK426-*ADH5*/pGK423-*THI3*
YA4K0	YPH499/pGK426-*ADH6*/pGK423
YA4K1	YPH499/pGK426-*ADH6*/pGK423-*kivd*
YA4K2	YPH499/pGK426-*ADH6*/pGK423-*ARO10*
YA4K3	YPH499/pGK426-*ADH6*/pGK423-*THI3*
YA5K0	YPH499/pGK426-*ADH7*/pGK423
YA5K1	YPH499/pGK426-*ADH7*/pGK423-*kivd*
YA5K2	YPH499/pGK426-*ADH7*/pGK423-*ARO10*
YA5K3	YPH499/pGK426-*ADH7*/pGK423-*THI3*
YA6K0	YPH499/pGK426-*SFA1*/pGK423
YA6K1	YPH499/pGK426-*SFA1*/pGK423-*kivd*
YA6K2	YPH499/pGK426-*SFA1*/pGK423-*ARO10*
YA6K3	YPH499/pGK426-*SFA1*/pGK423-*THI3*
Y06C250	YPH499/pGK406-*cimA*/pATP425
Y06C25C	YPH499/pGK406-*cimA*/pATP425-*leuC*(Cb)-*leuD*(Cb)
Y06C25E	YPH499/pGK406-*cimA*/pATP425-*leuC*(Ec)-*leuD*(Ec)
Y06C25 M	YPH499/pGK406-*cimA*/pATP425-*leuC*(Mj)-*leuD*(Mj)
Y26C250	YPH499/pGK426-*cimA*/pATP425
Y26C25C	YPH499/pGK426-*cimA*/pATP425-*leuC*(Cb)-*leuD*(Cb)
Y26C25E	YPH499/pGK426-*cimA*/pATP425-*leuC*(Ec)-*leuD*(Ec)
Y26C25 M	YPH499/pGK426-*cimA*/pATP425-*leuC*(Mj)-*leuD*(Mj)
Y5040	YPH499/pATP425/pATP424
Y5041	YPH499/pATP425/pATP424-*ILV1*
Y5042	YPH499/pATP425/pATP424-*tdcB*
Y5043	YPH499/pATP425/pATP424-*ilvA*
Y5C40	YPH499/pATP425-*cimA*-*leuC*(Cb)-*leuD*(Cb)/pATP424
Y5C41	YPH499/pATP425-*cimA*-*leuC*(Cb)-*leuD*(Cb)/pATP424-*ILV1*
Y5C42	YPH499/pATP425-*cimA*-*leuC*(Cb)-*leuD*(Cb)/pATP424-*tdcB*
Y5C43	YPH499/pATP425-*cimA*-*leuC*(Cb)-*leuD*(Cb)/pATP424-*ilvA*
Y5E40	YPH499/pATP425-*cimA*-*leuC*(Ec)-*leuD*(Ec)/pATP424
Y5E41	YPH499/pATP425-*cimA*-*leuC*(Ec)-*leuD*(Ec)/pATP424-*ILV1*
Y5E42	YPH499/pATP425-*cimA*-*leuC*(Ec)-*leuD*(Ec)/pATP424-*tdcB*
Y5E43	YPH499/pATP425-*cimA*-*leuC*(Ec)-*leuD*(Ec)/pATP424-*ilvA*
Y5M40	YPH499/pATP425-*cimA*-*leuC*(Mj)-*leuD*(Mj)/pATP424
Y5M41	YPH499/pATP425-*cimA*-*leuC*(Mj)-*leuD*(Mj)/pATP424-*ILV1*
Y5M42	YPH499/pATP425-*cimA*-*leuC*(Mj)-*leuD*(Mj)/pATP424-*tdcB*
Y5M43	YPH499/pATP425-*cimA*-*leuC*(Mj)-*leuD*(Mj)/pATP424-*ilvA*
B50	BY4741/pATP425
B5C	BY4741/pATP425-*cimA*-*leuC*(Cb)-*leuD*(Cb)
B5E	BY4741/pATP425-*cimA*-*leuC*(Ec)-*leuD*(Ec)
B5M	BY4741/pATP425-*cimA*-*leuC*(Mj)-*leuD*(Mj)
BG5C	BY4741Δ*GLY1*/pATP425-*cimA*-*leuC*(Cb)-*leuD*(Cb)
BG5E	BY4741Δ*GLY1*/pATP425-*cimA*-*leuC*(Ec)-*leuD*(Ec)
BG5M	BY4741Δ*GLY1*/pATP425-*cimA*-*leuC*(Mj)-*leuD*(Mj)
YG5040	YPH499Δ*GLY1*/pATP425/pATP424
YG5C42	YPH499Δ*GLY1*/pATP425-*cimA*-*leuC*(Cb)-*leuD*(Cb)/pATP424-*tdcB*
YG5E42	YPH499Δ*GLY1*/pATP425-*cimA*-*leuC*(Ec)-*leuD*(Ec)/pATP424-*tdcB*
YG5M42	YPH499Δ*GLY1*/pATP425-*cimA*-*leuC*(Mj)-*leuD*(Mj)/pATP424-*tdcB*
YG504030	YPH499Δ*GLY1*/pATP425/pATP424/pATP423
YG5C4231	YPH499Δ*GLY1*/pATP425-*cimA*-*leuC*(Cb)-*leuD*(Cb)/pATP424-*tdcB*/pATP423-*thrA*-*thrB*-*thrC*
YG5C4232	YPH499Δ*GLY1*/pATP425-*cimA*-*leuC*(Cb)-*leuD*(Cb)/pATP424-*tdcB*-*asd*/pATP423-*thrA*-*thrB*-*thrC*

**Table 2:** Plasmids used in this study

Plasmid	Description	Source of reference
pGK423	Yeast expression vector containing *PGK1* promoter, 2 μ origin, *HIS3* marker, no expression (control plasmid)	Ishii et al. [10]
pGK426	Yeast expression vector containing *PGK1* promoter, 2 μ origin, *URA3* marker, no expression (control plasmid)	Ishii et al. [10]
pGK406	Yeast integration vector containing *PGK1* promoter, *URA3* maker	Ishii et al. [10]
pATP425	Yeast three gene expression vector containing *ADH1*, *TDH3*, and *PGK1* promoters, 2 μ origin, *LEU2* marker, no expression (control plasmid)	
pATP424	Yeast three gene expression vector containing *ADH1*, *TDH3* and *PGK1* promoters, 2 μ origin, *TRP1* marker, no expression (control plasmid)	
pATP423	Yeast three gene expression vector containing *ADH1*, *TDH3* and *PGK1* promoters, 2 μ origin, *HIS3* marker, no expression (control plasmid)	
pGK423-*kivd*	*HIS3*, expression of *L. lactis kivd* gene	Kondo et al. [14]
pGK423-*ARO10*	*HIS3*, expression of *S. cerevisiae ARO10* gene	Kondo et al. [14]
pGK423-*THI3*	*HIS3*, expression of *S. cerevisiae THI3* gene	Kondo et al. [14]
pGK426-*ADH1*	*URA3*, expression of *S. cerevisiae ADH1* gene	Kondo et al. [14]
pGK426-*ADH2*	*URA3*, expression of *S. cerevisiae ADH2* gene	Kondo et al. [14]
pGK426-*ADH5*	*URA3*, expression of *S. cerevisiae ADH5* gene	Kondo et al. [14]
pGK426-*ADH6*	*URA3*, expression of *S. cerevisiae ADH6* gene	Kondo et al. [14]
pGK426-*ADH7*	*URA3*, expression of *S. cerevisiae ADH7* gene	Kondo et al. [14]
pGK426-*SFA1*	*URA3*, expression of *S. cerevisiae SFA1* gene	Kondo et al. [14]
pGK426-*cimA*	*URA3*, expression of *M. jannaschii cimA* gene	This study
pGK406-*cimA*	*URA3*, genomic integration of *M. jannaschii cimA* gene	This study
pATP425-*leuC*(Cb)-*leuD*(Cb)	*LEU2*, co-expression of *C. beijerinckii leuC* and *leuD* genes	This study
pATP425-*leuC*(Ec)-*leuD*(Ec)	*LEU2*, co-expression of *E. coli leuC* and *leuD* genes	This study
pATP425-*leuC*(Mj)-*leuD*(Mj)	*LEU2*, co-expression of *M. jannaschii leuC* and *leuD* genes	This study
pATP425-*cimA*-*leuC*(Cb)-*leuD*(Cb)	*LEU2*, co-expression of *M. jannaschii cimA*, *C. beijerinckii leuC* and *leuD* genes	This study
pATP425-*cimA*-*leuC*(Ec)-*leuD*(Ec)	*LEU2*, co-expression of *M. jannaschii cimA*, *E. coli leuC* and *leuD* genes	This study
pATP425-*cimA*-*leuC*(Mj)-*leuD*(Mj)	*LEU2*, co-expression of *M. jannaschii cimA*, *leuC* and *leuD* genes	This study
pATP424-*ILV1*	*TRP1*, expression of *S. cerevisiae ILV1* gene	This study
pATP424-*tdcB*	*TRP1*, expression of *E. coli tdcB* gene	This study
pATP424-*ilvA*	*TRP1*, expression of *E. coli ilvA* gene	This study
pATP424-*tdcB*-*asd*	*TRP1*, co-expression of *E. coli tdcB* and *asd* genes	This study
pATP423-*thrA*-*thrB*-*thrC*	*HIS3*, co-expression of *E. coli thrA*, *thrB* and *thrC* genes	This study

**Table 3:** Primers used in this study

Target gene	Primer (5′–3′)	Restriction enzyme
*cimA*	Fw; gggGGATCCatgatggtaaggatatttgatacaa	*Bam*HI
	Rv; cccCCCGGGttaattcaataacatattgattcct	*Xma*I
*cimA*	Fw; gggCCCGGGatgatggtaaggatatttgatacaa	*Xma*I
	Rv; cccGGCGCGCCttaattcaataacatattgattcct	*Asc*I
*leuC*(Cb)	Fw; gggGTCGACatgggaatgacaatgactcaaaaaa	*Sal*I
	Rv; cccCCCGGGCGGCCGCctacactaattcaggatcagttatt	*Not*I
*leuD*(Cb)	Fw; gggGTCGACCCTAGGatgagtgtaaaaggtaaagtattca	*Avr*II
	Rv; cccCCCGGGCCGGCCctatctatttcttatatatccaatc	*Fse*I
*leuC*(Ec)	Fw; gggGTCGACatggctaagacgttatacgaaaaat	*Sal*I
	Rv; cccCCCGGGCGGCCGCttatttaatgttgcgaatgtcggcg	*Not*I
*leuD*(Ec)	Fw; gggGTCGACCCTAGGatggcagagaaatttatcaaacaca	*Avr*II
	Rv; cccCCCGGGCCGGCCttaattcataaacgcaggttgtttt	*Fse*I
*leuC*(Mj)	Fw; gggGTCGACatgggaatgacaattgtagagaaga	*Sal*I
	Rv; cccCCCGGGCGGCCGCttataaatcccttgggtcaacaagt	*Not*I
*leuD*(Mj)	Fw; gggGTCGACCCTAGGatgagaagtataataaagggaagag	*Avr*II
	Rv; cccCCCGGGCCGGCCttattggctttcagccatctttttc	*Fse*I
*ILV1*	Fw; gggGTCGACatgtcagctactctactaaagcaac	*Sal*I
	Rv; cccGGATCCGCGGCCGCttaatatttcaagaatttttgataa	*Not*I
*tdcB*	Fw; gggGTCGACatgcatattacatacgatctgccgg	*Sal*I
	Rv; cccGGATCCGCGGCCGCttaagcgtcaacgaaaccggtgatt	*Not*I
*ilvA*	Fw; gggGTCGACatggctgactcgcaacccctgtccg	*Sal*I
	Rv; cccGGATCCGCGGCCGCctaacccgccaaaaagaacctgaac	*Not*I
*asd*	Fw; CCTAGGatgaaaaatgttggttttatcggctggcgc	*Avr*II
	Rv; GGCCGGCCttacgccagttgacgaagcatccgacgcag	*Fse*I
*thrA*	Fw; GTCGACatgcgagtgttgaagttcggcggtacatca	*Sal*I
	Rv; GCGGCCGCtcagactcctaacttccatgagagggtacg	*Not*I
*thrB*	Fw; CCTAGGatggttaaagtttatgccccggcttccagt	*Avr*II
	Rv; GGCCGGCCttagttttccagtactcgtgcgcccgccgt	*Fse*I
*thrC*	Fw; CCCGGGatgaaactctacaatctgaaagatcacaat	*Xma*I
	Rv; GGCGCGCCttactgatgattcatcatcaatttacgcaa	*Asc*I
*GLY1*	Fw; TCACTTGCCATATTCGTTCACCGGTTTTTCTTTTTATTTC	
	Rv; caatctgctctgatgccgcatagttaagccACAAAAACCCTAACAATACACATGATGCAACTGGAACGCATGTGTTTATGTTTGCGTTTGTGTGCGGGAG	
*URA3*	Fw; TGTATTGTTAGGGTTTTTGTggcttaactatgcggcatcagagcagattg	
	Rv; GAAAAAAAGGAAGAGGGTAGCAATCCTAAAACAAAAACCCTAACAATACACATGATGCAACTGGAACGCAttagttttgctggccgcatcttctcaaata	

### Deletion of competing pathway

*GLY1* was disrupted according to the method of Akada et al. [13]. Briefly, 300 bp of the 5′-flank of *GLY1* was PCR amplified with a standard forward primer, and a reverse primer containing a 20 bp sequence of the 5′-flank followed by 40 bp sequence of the 3′-flank of *GLY1*. Separately, the *URA3* marker cassette of pGK426 was PCR amplified with a forward primer containing a 20 bp overlap of the former PCR product and a reverse primer containing a 70 bp sequence of the 3′-flank of *GLY1*. Both amplified fragments were mixed and combined by PCR. The final PCR product was introduced into YPH499 using the lithium acetate method and the correctly integrated transformant was selected. The *URA3* marker was then eliminated by counter selection with 5-fluoroorotic acid. Disruption of *GLY1* and elimination of *URA3* was confirmed by diagnostic PCR to check fragment sizes. The constructed strain, which has Δ*GLY1* allele, was designated YPH499 Δ*GLY1*. Subsequently, double deletion strains with Δ*GLY1* and other (Δ*ARO4*, Δ*ALT1*, ΔILV6, Δ*CIT1* or Δ*MET2*) were constructed in common with deletion of *GLY1*.

### Fermentation of engineered strains

The transformants were cultured for 48 h at 30 °C in 5 mL of SD minimal medium (6.7 g/L yeast nitrogen base without amino acids and 20 g/L glucose) containing the required amino acids. Following centrifugation at 3000 rpm for 5 min and removal of the supernatant, yeast cells were cultured in 5 mL of fresh SD minimal medium containing the required amino acids with/without 8 g/L 2 KB. The concentration of 1-propanol in the medium 72 h after the start of fermentation was determined using GC–MS (GCMS-QP2010 Plus; Shimadzu) following a previously described procedure [14].

For oxygen-limited fermentation, yeast transformants were anaerobically cultivated in SD minimal medium containing the required amino acids for 48 h at 30 °C. The cells were collected by centrifugation at 1000*g* for 5 min at 4 °C and washed twice with sterile water. The cells were then placed in 50 mL of SD minimal medium. The initial cell concentration was adjusted to OD_600_ = 20. All fermentations were performed at 30 °C with mild agitation in 100 mL closed bottles equipped with a bubbling CO_2_ outlet.

## Results and discussion

### Overexpression of 2-keto acid decarboxylase and alcohol/aldehyde dehydrogenase

**Fig. 2:**
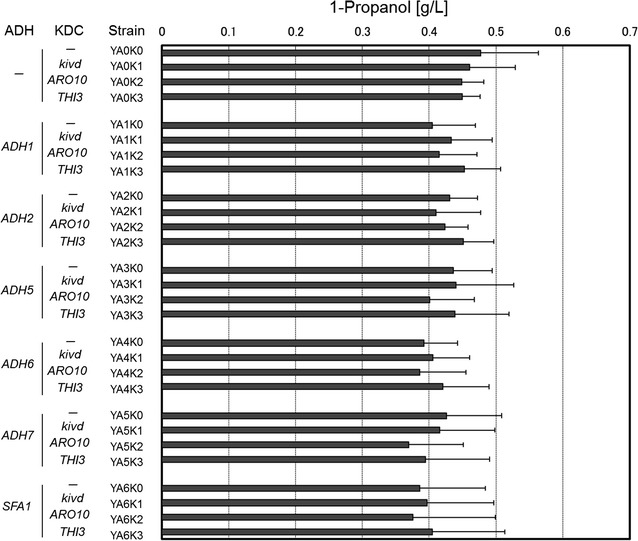
Production of 1-propanol from added 2 KB in various KDC- and ADH-overexpressing *S. cerevisiae* YPH499 strains

It has been reported that various alcohols can be made from α-keto acids by two-step catalytic reactions with 2-ketoacid decarboxylase (KDC) and alcohol/aldehyde dehydrogenase (ADH) [5]. Thus, 1-propanol can be produced from 2 KB that is the intermediate metabolite of isoleucine biosynthesis (Fig. 1a). In this study, we first examined the KDC and ADH enzymes that efficiently convert 2 KB to 1-propanol in *S. cerevisiae* (Fig. 2). We chose three KDC enzymes (phenylpyruvate decarboxylase, *ARO10I*, and alpha-ketoisocaproate decarboxylase, *THI3*, derived from *S. cerevisiae*; and α-ketoisovalerate decarboxylase, *Kivd*, derived from *Lactococcus lactis*) and six ADH enzymes (*ADH1*, *2*, *5*, *6*, *7*, and *SFA1*, derived from *S. cerevisiae*), in reference to a previous report [14], for overexpression in *S. cerevisiae*.

The genes encoding these KDC and ADH enzymes were co-introduced into the YPH499 yeast strain in all possible combinations, and the transformants were fermented in SD selective media containing 8 g/L of 2 KB for 72 h. We found that all transformants showed similar productivities for 1-propanol (approx. 400 mg/L) (Fig. 2). The fact that overexpression of KDC and ADH in *S. cerevisiae* provided no advantage for the production of 1-propanol, indicates either that the selected enzymes did not have specific activity for the conversion of 2 KB into 1-propanol, or that endogenous yeast KDC and ADH enzymes already provide sufficient activity for this purpose. Given that the negative control strain (YA0K0; exogenously overexpressing neither KDC nor ADH) also produced 1-propanol, the latter is most probable. Indeed, engineered *E. coli* overexpressing *ARO10* (from *S. cerevisiae*) or *Kivd* (from *L. lactis*) with *ADH2* (from *S. cerevisiae*) have been shown to exhibit the activity required to convert 2 KB into 1-propanol [5].

### Construction of a 2-ketobutyrate biosynthesis pathway via citramalate

Since *S. cerevisiae* appears to have sufficient KDC and ADH activity to convert 2 KB into 1-propanol, we next tried engineering yeast metabolic pathways to increase levels of 2 KB, the precursor of 1-propanol, using glucose as a carbon source. In *E. coli*, 2 KB is normally produced through the enzymatic conversion of threonine by threonine dehydratase. Engineered *E. coli* with increased 1-propanol productivity have been developed by introducing an artificial pathway via citramalate, which can convert pyruvate into 2 KB, in addition to the original threonine-mediated pathway (Fig. 1b) [15, 16]. Since *E. coli* has endogenous genes encoding citramalate hydrolyase (*leuC* and *leuD*) and 3-isopropylmalate dehydrogenase (*leuB*), the citramalate-mediated pathway can been completed by artificially expressing the citramalate synthase (*cimA*) gene derived from *Methanococcus jannaschii* (Mj). In *S. cerevisiae*, 2 KB is also produced endogenously via threonine (Fig. 1a), however, it does not carry the corresponding genes for citramalate synthase (*cimA*) or citramalate hydrolyase (*leuC* and *leuD*). Drawing on the experience from *E. coli*, we therefore constructed an artificial citramalate-mediated pathway to overproduce 2 KB from pyruvate and examined the productivity of 1-propanol in *S. cerevisiae* (Fig. 1b).

Although *S. cerevisiae* has an endogenous *LEU2* gene that encodes 3-isopropylmalate dehydrogenase (encoded as *leuB* in *E. coli*), the laboratory yeast strains (YPH499 and BY4741) used in this study lack the functional *LEU2* gene, as they are auxotrophs for the purposes of selection after gene transfection. Therefore, we used an expression plasmid carrying the *LEU2* auxotrophic marker to compensate for 3-isopropylmalate dehydrogenase activity. For citramalate hydrolyase, we selected *leuC* (citramalate hydrolyase, large subunit) and *leuD* (small subunit) genes from three different sources: thermophilic methanogenic archaea, *M. jannaschii* (Mj), gram-negative and facultative anaerobic bacteria, *E. coli* (Ec), and the gram-positive and obligate anaerobe *Clostridium beijerinckii* (Cb). These gene pairs were introduced into the autonomously-replicating plasmid harboring the *LEU2* marker. For citramalate synthase, which catalyzes 2 KB biosynthesis via citramalate from the central metabolite pyruvate, we used the *cimA* gene derived from *M. jannaschii*, which was successfully used in *E. coli* in the previous study [15, 16]. Two methods of expressing *cimA* were tested, the first being a single-copy genomic integration into the *ura3* locus to stabilize gene replication, and the second being co-integration into the *LEU2* marker plasmid along with *leuC* and *leuD*, in order to increase overall expression.

**Fig. 3:**
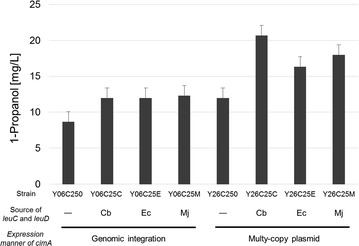
Production of 1-propanol in *S. cerevisiae* YPH499 strains expressing an artificially engineered pathway from pyruvate to 2 KB. (Mj: *M. jannaschii*, Ec: *E. coli*, Cb: *Clostridium beijerinckii*)

In fermentation using the engineered YPH499 strains (Table 1) in SD medium (20 g/L glucose) without 2 KB, the use of the multi-copy plasmid expressing *cimA* resulted in higher production of 1-propanol than direct genomic integration, in all cases tested (Fig. 3). This indicated that high expression of *cimA* is more successful for 1-propanol production in yeast. Comparing the biological source of *leuC* and *leuD*, Cb-derived genes showed the highest productivity of 1-propanol (Fig. 3). Yeast with genomic integration of *cimA* that were transfected with mock (non-*LEU2* expressing) plasmid (Y06C250) produced 8.7 mg/L of 1-propanol (Fig. 3), while the engineered strain with plasmid-driven expression of *cimA* and Cb-derived *leuC* and *leuD* (Y26C25C) produced a much higher level of 1-propanol (20.7 mg/L). These results suggested that the exogenous expression of *cimA*, *leuC* and *leuD* (and the *LEU2* marker) allowed 2 KB biosynthesis in *S. cerevisiae* via citramalate, as has previously been shown for *E. coli*.

### Overexpression of threonine dehydratase

**Fig. 4:**
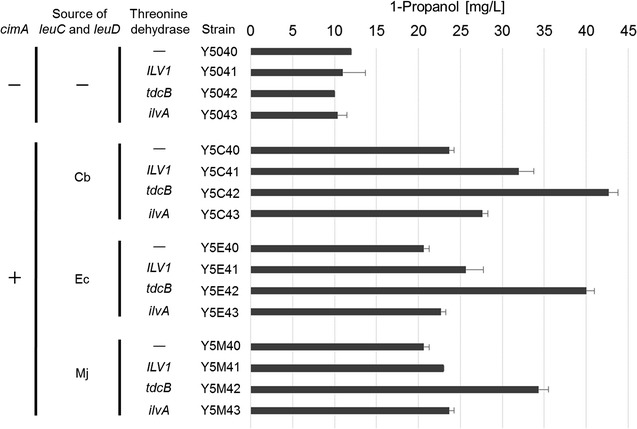
Production of 1-propanol in *S. cerevisiae* YPH499 strains expressing an artificially engineered pathway from pyruvate to 2 KB and overexpression of genes encoding the enzyme threonine dehydratase. (Mj: *M. jannaschii*, Ec: *E. coli*, Cb: *Clostridium beijerinckii*)

To further increase the production of 1-propanol, we attempted to enhance the endogenous threonine-mediated pathway for 2 KB biosynthesis as shown in Fig. 1c. To do this we overexpressed threonine dehydratase, which catalyzes the conversion of threonine to 2 KB. Three types of threonine dehydratase gene were tested, namely, *ILV1* from *S. cerevisiae*, *tdcB* from *E. coli*, and *ilvA* also from *E. coli* (Table 1 and Fig. 4). We found that strains overexpressing threonine dehydratase in the absence of *cimA*, *leuC* and *leuD* (Y5041 ~ 3) showed no significant increase in 1-propanol production (Fig. 4). However, all strains overexpressing threonine dehydratase in conjunction with *cimA*, *leuC* and *leuD* (Y5C41 ~ 3, Y5E41 ~ 3 and Y5M41 ~ 3) showed an increase in 1-propanol production, with the expression of *tdcB* (*E. coli*) having the most significant impact. The Y5C40 strain expressing *cimA* and Cb-derived *leuC* and *leuD* produced 23.7 mg/L of 1-propanol, while Y5C42 (representing the Y5C40 strain with co-expression of *tdcB*) produced 42.7 mg/L of 1-propanol. Although it is unclear why the overexpression of threonine dehydratase alone (Y5041 ~ 3) resulted in no improvement, we thought there might be no extra threonine, competing pathway from threonine to glycine might be strong or coenzyme balance might affect this result due to the reaction of LEU2 enzyme required for coenzyme. However, the findings clearly demonstrate the synergistic effect of threonine dehydratase expression and citramalate-mediated 2 KB biosynthesis for 1-propanol production in yeast.

### Deletion of competing biochemical pathways

Next, we attempted to increase the production of 1-propanol by decreasing carbon flux into competing pathways for 2 KB and amino-acid metabolism. To do this, we used yeast strains with deletion of specific biochemical pathways from a single gene deletion library of BY4741. As shown in Fig. 1a, *ILV2*, *ILV3*, *ILV5* and *BAT1* are candidate target genes for the knockout of the biosynthetic pathway for valine, leucine and isoleucine, however there was no strain with deletion of these genes in the library. This indicates that the deletion of each of these genes is either lethal or results in poor growth, ruling out these genes as candidates for deletion in our study.

**Fig. 5:**
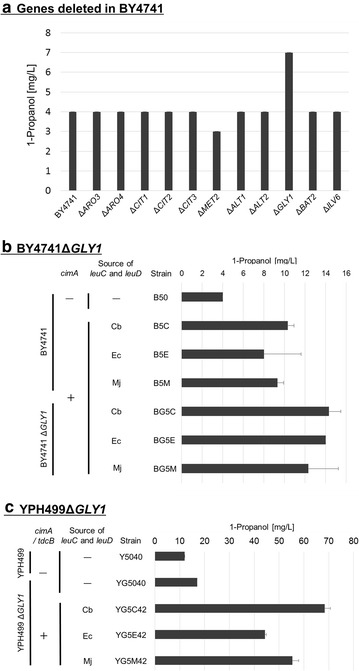
Deletion of metabolic pathways competing with 1-propanol production in yeast. **a** 1-Propanol production in strains from a single gene deletion library of BY4741. **b** Comparison of BY4741 with BY4741Δ*GLY1.* **c** Comparison of YPH499 with YPH499Δ*GLY1.* (Mj: *M. jannaschii*, Ec: *E. coli*, Cb: *Clostridium beijerinckii*)

Of the remaining candidates, 11 genes were selected for targeting (colored green in Fig. 1a). *ARO3* and *ARO4* encode 3-deoxy-d-arabino-heptulosonate-7-phosphate synthase from the tryptophan and phenylalanine biosynthetic pathway. *ALT1* and *ALT2* encode alanine transaminase from the alanine biosynthetic pathway. *CIT1*, *CIT2* and *CIT3* encode citrate synthase from the glutamine, arginine and lysine biosynthetic pathway. *MET2* encodes l-homoserine-*O*-acetyltransferase from the methionine biosynthetic pathway. *GLY1* encodes threonine aldolase, which converts threonine to glycine. *ILV6* encodes the regulatory subunit of acetolactate synthase contained in the valine, leucine and isoleucine biosynthetic pathway. *BAT2* encodes the branched-chain amino acid transaminase. Using BY4741 strains with individual deletions of each of these genes, we compared 1-propanol production in YPD rich medium (Fig. 5a). We found that only the strain deleting *GLY1* (encoding threonine aldolase) showed an increase in 1-propanol production (Fig. 5a). Since Gly1 constitutes the main pathway to produce glycine from threonine in yeast [17], the deletion of *GLY1* would decrease the loss of threonine and increase its conversion to 2 KB. *GLY1* is the sole gene that encodes threonine aldolase, and the absence of isozymes no doubt enhances the efficacy of this approach. To test whether the *GLY1*-deleted strain could increase the production of 1-propanol via citramalate, we introduced *cimA*, *leuC*, and *leuD* into BY4741Δ*GLY1* (Fig. 5b). Following fermentation using SD media, BY4741Δ*GLY1* strains with *cimA*, *leuC*, and *leuD* (BG5C, BG5E, and BG5M) had higher productivity of 1-propanol than original BY4741 strains with *cimA*, *leuC*, and *leuD* (B5C, B5E and B5M). This result indicated that the *GLY1* deletion could indeed fulfill the function of increasing 1-propanol production from the artificial citramalate pathway.

Comparing Fig. 5b with Fig. 3, it is clear that production of 1-propanol in YPH499 was higher than that of BY4741. Therefore, we subsequently constructed a YPH499Δ*GLY1* strain to enhance 2 KB biosynthesis via both the citramalate and threonine pathways. As shown in Fig. 5c, YPH499Δ*GLY1* (YG5040) demonstrated higher production of 1-propanol than wildtype YPH499 (Y5040). Furthermore, YPH499Δ*GLY1* with expression of *cimA*, *leuC*, *leuD*, and *tdcB* (YG5C42) produced 68.3 mg/L of 1-propanol (Fig. 5c), whereas YPH499 harboring the same genes (Y5C42) produced 42.6 mg/L (Fig. 4). Thus, just as in BY4741, the deletion of *GLY1* enhanced the production of 1-propanol in YPH499 yeast strains with modifications of both the citramalate and threonine pathways.

### Overexpression of threonine synthase

**Fig. 6:**
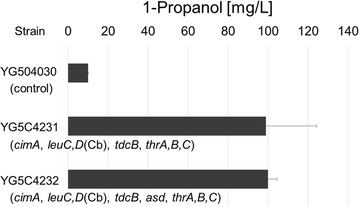
Production of 1-propanol in *S. cerevisiae* YPH499Δ*GLY1* strains with an artificially engineered pathway from pyruvate to 2 KB and overexpression of *tdcB* and genes encoding for threonine synthase. (Cb: *Clostridium beijerinckii*)

To further improve the production of 1-propanol, we also aimed to enhance the threonine biosynthetic pathway via aspartate as shown Fig. 1d. To increase the carbon flux from aspartate to threonine, we selected four genes (bifunctional *thrA*, encoding aspartokinase and homoserine dehydrogenase I; *thrB*, encoding homoserine kinase; *thrC*, encoding threonine synthase; and *asd*, encoding aspartate-semialdehyde dehydrogenase) derived from *E. coli* [15]. In addition to *cimA*, *leuC*, *leuD*, and *tdcB*, these four genes (*thrA*, *thrB*, *thrC*, and *asd*) or alternatively just three genes (*thrA*, *thrB* and *thrC*), were introduced into the YPH499Δ*GLY1* strain to generate YG5C4232 and YG5C4231, respectively. Following fermentation in SD media, both YG5C4231 and YG5C4232 produced ~ 100 mg/L of 1-propanol, with the presence or absence of the *asd* gene thus appearing to make little difference (Fig. 6). Compared to YPH499Δ*GLY1* expressing *cimA*, *leuC*, *leuD*, and *tdcB* (YG5C42; 68.3 mg/L in Fig. 5b), these strains therefore demonstrated an additional increase in 1-propanol production. This indicates that enhancement of aspartate-mediated threonine biosynthesis co-operates with the *GLY1* deletion in regard to enhancement of 1-propanol production via the citramalate, threonine, and 2 KB pathways.

### Oxygen-limited fermentation of engineered strains

**Fig. 7:**
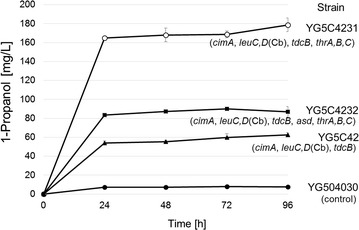
Oxygen-limited fermentation of engineered YPH499Δ*GLY1* strains. (Cb: *Clostridium beijerinckii*)

Finally, we measured the time course for 1-propanol production of engineered strains in 50 mL of SD medium (initial cell concentration, OD_600_ = 20) under oxygen-limited condition using fermentation bottles (Fig. 7). We found that the strains YG504030 (YPH499Δ*GLY1*; control), YG5C42 (*cimA*/*leuC*, *leuD*/*tdcB*) and YG5C4232 (*cimA*/*leuC*, *leuD*/*tdcB*/*thrA*, *B*, *C*/*asd*) showed a similar production of 1-propanol under limited oxygen conditions compared to their growth in test tubes. In contrast, YG5C4231 (*cimA*/*leuC*, *leuD*/*tdcB*/*thrA*, *B*, *C*) displayed an approximately two-fold higher productivity compared under these conditions (Fig. 7). This result suggests that the threonine biosynthetic pathway via aspartate is enhanced during oxygen-limited fermentation, resulting in yet greater 1-propanol production. We eventually obtained 179 mg/L of 1-propanol from 20 g/L of glucose using YG5C4231 (*cimA*/*leuC*, *leuD*/*tdcB*/*thrA*, *B*, *C*) under oxygen-limited conditions, the highest level of production observed for any of the 1-propanol-producing yeast strains. As the reason of little change from 24 to 96 h, we thought the glucose was exhausted for 24 h due to high concentration of the initial added yeast.

## Conclusions

In the present study, we modified metabolic pathways of *S. cerevisiae* to engineer yeast strains producing 1-propanol. Firstly, we observed that the activity of endogenous yeast KDC and ADH is sufficient to convert 2 KB to 1-propanol. Secondly, we found that 1-propanol production could be increased by constructing an artificial 2 KB biosynthetic pathway from pyruvate via citramalate, with the introduction of *cimA* and *leuC/leuD* genes from *M. jannaschii* and *C. beijerinckii*, respectively. Furthermore, in addition to the overexpression of threonine dehydratase (with the introduction of *tdcB*), and enhancement of threonine biosynthesis from aspartate (with the introduction of *thrA*, *thrB* and *thrC*), 1-propanol production was greatly increased by deletion of the *GLY1* gene that regulates a competing pathway converting threonine to glycine. While the control YPH499 strain (Y5040) produced only 12 mg/L of 1-propanol in test tubes, the engineered strain YG5C4231 produced 99 mg/L. Moreover, in the context of high-density anaerobic fermentation, we succeeded in producing 179 mg/L of 1-propanol using this strain. These results demonstrate that construction of a citramalate-mediated pathway as the production method of 1-propanol in *S. cerevisiae* is effective. For yet further improvement of 1-propanol production in *S. cerevisiae*, it may be necessary to engineer the carbon flux from ethanol to 2 KB and oxidoreduction balance due to coenzyme. For example, as shown in Additional file 2 using yeast strains of Additional file 3, double deletion of competing pathway have the potential to increase 1-propanol production.

## Additional files

**Additional file 1.** Primers used for the construction of double deletion strains in Additional file 2.

**Additional file 2.** Double deletion of metabolic pathways competing with 1-propanol production in YPH499.

**Additional file 3.** Yeast strains used in Additional file 2.
