# Supplementary material for: Metabolic engineering of the 2-ketobutyrate biosynthetic pathway for 1-propanol production in Saccharomyces cerevisiae
Source: Microb Cell Fact. 2018 Mar 9;17:38. doi: 10.1186/s12934-018-0883-1 (PMC5844117; doi:10.1186/s12934-018-0883-1)
Supplement: Supplementary file 1 — Additional file 1. Primers used for the construction of double deletion strains in Additional file 2. [file 12934_2018_883_MOESM1_ESM.docx]

**Additional file 1.** Primers used for the construction of double deletion strains in Additional file 2.

| Target gene | Primer (5′-3′) |
| --- | --- |
| *ARO4* | Fw; GAAATGAAAAAATTTTGCTTGAAATTTTGAAAAAAAGATG |
|  | Rv; actctcagtacaatctgctctgatgccgcatagttaagccAGAGGAAAGAATGTACGTTACATATATCATTAAAAAAACATTCTGATAGATTCTTTTGTT |
| *ARO4URA3* | Fw; TAACGTACATTCTTTCCTCTggcttaactatgcggcatca |
|  | Rv; AGCGCTAAACACAATTAAATAATACCGAATTGGCAGTGGTAGAGGAAAGAATGTACGTTACATATATCATTAAAAAAACAttagttttgctggccgcatc |
| *ALT1* | Fw; CTAACGATTTTCGACCTTTCGTTGAGACAACTTCACGTAC |
|  | Rv; actctcagtacaatctgctctgatgccgcatagttaagccTATTTAAATGTTTATTGAAGACTGTTCTGCCCCCTTTTATCGTGGAGAGAGCAAAATAAA |
| *ALT1URA3* | Fw; CTTCAATAAACATTTAAATAggcttaactatgcggcatca |
|  | Rv; GTGGAAGAAAGAAACGATCACATTATTATAATAAACTAGCTATTTAAATGTTTATTGAAGACTGTTCTGCCCCCTTTTATttagttttgctggccgcatc |
| *ILV6* | Fw; TACGCTCTCACGGACACCATTGAGTGCTTATCTAGGATGC |
|  | Rv; actctcagtacaatctgctctgatgccgcatagttaagccAGGAGAGTCCCGAGGGCGATCGCAAGGCCGAGAGACTAACTAGAAAGACTGGGTTAGTGA |
| *ILV6URA3* | Fw; ATCGCCCTCGGGACTCTCCTggcttaactatgcggcatca |
|  | Rv; AATAGGCTCATGGTTATAGATACGTTATATAGATGTATAGAGGAGAGTCCCGAGGGCGATCGCAAGGCCGAGAGACTAACttagttttgctggccgcatc |
| *CIT1* | Fw; TTGTGTTATTGGAGGATCGCAATCCCTTTGGAGCTTTTCC |
|  | Rv; actctcagtacaatctgctctgatgccgcatagttaagccTTGAATAGTCGCATACCCTGAATCAAAAATCAAATTTTCCCTTCGTAAATAGTATTATAT |
| *CIT1URA3* | Fw; CAGGGTATGCGACTATTCAAggcttaactatgcggcatca |
|  | Rv; ATAAATTATAAACTACTCATTCGTATATGAAAATACGTGTTTGAATAGTCGCATACCCTGAATCAAAAATCAAATTTTCCttagttttgctggccgcatc |
| *MET2* | Fw; GGATGCACTCATTTGATAGTAAACTAAGTCATGTTAATCG |
|  | Rv; actctcagtacaatctgctctgatgccgcatagttaagccTTTATCTATGTTATGCCTGAGGTATGTGTGGTATCTATCCTCCTTTTATACTACGTTTTT |
| *MET2URA3* | Fw; TCAGGCATAACATAGATAAAggcttaactatgcggcatca |
|  | Rv; TGTCTATATATAAATATAGATATAGATATACATGTACTGGTTTATCTATGTTATGCCTGAGGTATGTGTGGTATCTATCCttagttttgctggccgcatc |
