# Supplementary figures and images for: Metabolic engineering of the 2-ketobutyrate biosynthetic pathway for 1-propanol production in Saccharomyces cerevisiae
Source: Microb Cell Fact. 2018 Mar 9;17:38. doi: 10.1186/s12934-018-0883-1 (PMC5844117; doi:10.1186/s12934-018-0883-1)

## Slide 1
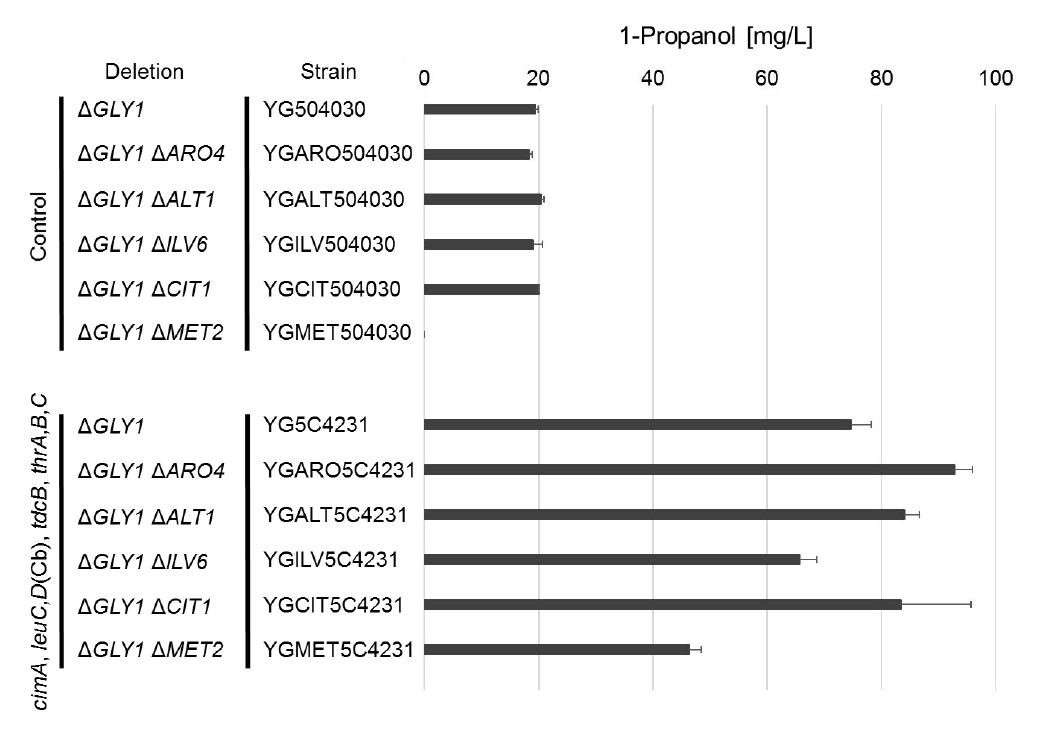

Supplement: Supplementary file 2 — Additional file 2. Double deletion of metabolic pathways competing with 1-propanol production in YPH499. [file 12934_2018_883_MOESM2_ESM.pptx]
