# Supplementary material for: Metabolic engineering of the 2-ketobutyrate biosynthetic pathway for 1-propanol production in Saccharomyces cerevisiae
Source: Microb Cell Fact. 2018 Mar 9;17:38. doi: 10.1186/s12934-018-0883-1 (PMC5844117; doi:10.1186/s12934-018-0883-1)
Supplement: Supplementary file 3 — Additional file 3. Yeast strains used in Additional file 2. [file 12934_2018_883_MOESM3_ESM.docx]

**Additional file 3.** Yeast strains used in Additional file 2.

| Strains | Genotypes |
| --- | --- |
| YGARO504030 | YPH499Δ*GLY1*Δ*ARO4* / pATP425 / pATP424 / pATP423 |
| YGARO5C4231 | YPH499Δ*GLY1*Δ*ARO4* / pATP425-*cimA*-*leuC*(Cb)-*leuD*(Cb)  / pATP424-*tdcB* / pATP423-*thrA*-*thrB*-*thrC* |
| YGALT504030 | YPH499Δ*GLY1*Δ*ALT1* / pATP425 / pATP424 / pATP423 |
| YGALT5C4231 | YPH499Δ*GLY1*Δ*ALT1* / pATP425-*cimA*-*leuC*(Cb)-*leuD*(Cb)  / pATP424-*tdcB* / pATP423-*thrA*-*thrB*-*thrC* |
| YGILV504030 | YPH499Δ*GLY1*Δ*ILV6* / pATP425 / pATP424 / pATP423 |
| YGILV5C4231 | YPH499Δ*GLY1*Δ*ILV6* / pATP425-*cimA*-*leuC*(Cb)-*leuD*(Cb)  / pATP424-*tdcB* / pATP423-*thrA*-*thrB*-*thrC* |
| YGCIT504030 | YPH499Δ*GLY1*Δ*CIT1* / pATP425 / pATP424 / pATP423 |
| YGCIT5C4231 | YPH499Δ*GLY1*Δ*CIT1* / pATP425-*cimA*-*leuC*(Cb)-*leuD*(Cb)  / pATP424-*tdcB* / pATP423-*thrA*-*thrB*-*thrC* |
| YGMET504030 | YPH499Δ*GLY1*Δ*MET2* / pATP425 / pATP424 / pATP423 |
| YGMET5C4231 | YPH499Δ*GLY1*Δ*MET2* / pATP425-*cimA*-*leuC*(Cb)-*leuD*(Cb)  / pATP424-*tdcB* / pATP423-*thrA*-*thrB*-*thrC* |
